# Effect of Aqueous Extract of *Massularia acuminata* Stem on Sexual Behaviour of Male Wistar Rats

**DOI:** 10.1155/2011/738103

**Published:** 2011-01-02

**Authors:** M. T. Yakubu, M. A. Akanji

**Affiliations:** Phytomedicine, Toxicology, Reproductive and Developmental Biochemistry Research Laboratory, Department of Biochemistry, University of Ilorin, PMB 1515, Ilorin, Nigeria

## Abstract

Ancient literature alluded to the use of a number of plants/preparations as sex enhancer. One of such botanicals is *Massularia acuminata* in which the stem has been acclaimed to be used as an aphrodisiac. Documented experiments or clinical data are, however, lacking. Therefore, this study was undertaken to evaluate the acclaimed aphrodisiac activity of *M. acuminata* stem. Sixty male rats were completely randomized into 4 groups (A–D) of 15 each. Rats in group A (control) were administered with 1 mL of distilled water (the vehicle) while those in groups B, C, and D were given same volume containing 250, 500, and 1000 mg/kg body weight of the extract, respectively. Sexual behaviour parameters were monitored in the male rats for day 1 (after a single dose), day 3 (after three doses, once daily), and day 5 (after five doses, once daily) by pairing with a receptive female (1 : 1). The male serum testosterone concentration was also determined. Cage side observation on the animals revealed proceptive behaviour (ear wiggling, darting, hopping, and lordosis) by the receptive female rats and precopulatory behaviour (chasing, anogenital sniffing and mounting) by the extract-treated male rats. The extract at 500, and 1000 mg/kg body weight significantly (*P* < .05) increased the frequencies of mount and intromission. In addition, the ejaculation latency was significantly prolonged (*P* < .05). The latencies of mount and intromission were reduced significantly whereas ejaculation frequency increased. The extract also reduced the postejaculatory interval of the animals. Computed percentages of index of libido, mounted, intromitted, ejaculated and copulatory efficiency were higher in the extract treated animals compared to the distilled water-administered control whereas the intercopulatory interval decreased significantly. The extract also significantly (*P* < .05) increased the serum testosterone content of the animals except in those administered with 250 mg/kg body weight on days 1 and 3. Data from this study identified that the aqueous extract of *Massularia acuminata* stem enhanced sexual behaviour in male rats. The improved sexual appetitive behaviour in male rats at the doses of 500 and 1000 mg/kg body weight of *Massularia acuminata* stem may be attributed, at least in part, to the alkaloids, saponins, and/or flavonoids since these phytochemicals has engorgement, androgen enhancing, and antioxidant properties.

## 1. Introduction

The main essence of marriage in humans is procreation and/or sexual fulfillment of both partners that is initiated by the mating of a male with a female in sexual intercourse. For there to be a normal sexual intercourse in males, the sexual organs and factors relating to erection of the copulatory organ must function normally. The repeated inability of the male to perform this function, at least effectively, or a disorder that interfere with his full sexual response cycle is termed male sexual dysfunction (MSD) [1]. MSD is common worldwide among men of all ages, ethnicities, and cultural backgrounds. Although MSD rarely threatens physical health, it can take a heavy psychological toll, bringing on depression, anxiety, and debilitating feelings of inadequacy. Sexual dysfunction in men takes different forms, such as disorders of desire (persistently or recurrently deficient sexual fantasy and desire for sexual activity), disorders of orgasm (persistent or recurrent delay in, or absence of, orgasm after a normal sexual excitement phase), erectile dysfunction (persistent failure to generate sufficient penile body pressure to achieve vaginal penetration and/or the inability to maintain this degree of penile rigidity until ejaculation), disorders of ejaculation (persistent or recurrent ejaculation with minimum sexual stimulation that occurs before, upon, or shortly after penetration and before a person wishes it or a situation where ejaculation does not occur at all) and failure of detumescence (prolonged priapism lasting for more than 4 h). MSD is of varied etiologies and these include personal life styles (chronic alcohol abuse, cigarette smoking), androgen deficiency, aging population, psychological disorders, side effects of some antihypertensives, central agents, psychiatric medications, antiulcer, antidepressants, and antiandrogens and chronic medical conditions like diabetes, hypertension and pulmonary cancer [2]. 

 The incidence of sexual inadequacy in human males has led to the development of a number of available treatment options. Unfortunately however, these options are too expensive, not easily accessible and with some serious side effects such as aching in the penis, urethral burning, infection, pains and bleeding [2]. This problem, coupled with the increasing number of men seeking help for the treatment of MSD, has necessitated the need for more pharmacological research on cheaper and natural treatment options for this menace [3]. Plant-derived chemicals that have sex-enhancing potentials in animals have received a great deal of attention and have become known worldwide as an instant treatment [4]. These phytochemicals increase libido (sexual desire and arousal), sexual potency (effectiveness of erection) and or sexual pleasure [5]. For example, *Syzygium aromaticum, Montanoa tomentosa and Fadogia agrestis *have been reported to have sexual function enhancing effects in male rats [6–8]. The aphrodisiac activity of these plants has been attributed to one or more of the phytoconstitutents. For example, sterols, phenols, alkaloids and amino acids in *Myristica fragrans *Houtt. (nutmeg) have been suggested to be responsible for improving sexual function through changes in neurotransmitter level [9] whereas saponins in *Panax ginseng *act as a nitric oxide donor and induce the relaxation of smooth muscle of rabbit *Corpus cavernosum* through the L arginine/nitric oxide pathway [10]. In addition, alkaloids (keayanidine B and keayanine) in *Microdesmis keayana *roots have been reported to enhance sexual behaviour in male rats by increasing the production of nitric oxide [11]. Although, several plants and their bioactive principle(s) have been screened for sex enhancing effect, the continued search for new botanicals with aphrodisiac activity is still attractive because they are readily accessible, affordable and les toxic. *Mussularia acuminata* is one of such plant claimed to have sex enhancing effect in the folk medicine of Nigeria with no experimental or clinical data in the open scientific literature.

*Massularia acuminata* (G. Don) Bullock ex Hoyl. (Rubiaceae) known as *pako ijebu* or *orin ijebu* (Yoruba-Western Nigeria), is a tree growing up to 5 m high. It is distributed from Sierria Leone through Nigeria to Democratic Republic of Congo. The large leaves are practically stalkless, elliptic, acuminate and almost glaborious. Phytoconstituents of the aqueous extract of *M. acuminata *stem included alkaloids (0.22%), saponins (1.18%), anthraquinones (0.048%), flavonoids (0.032%), tannins (0.75%) and phenolics (0.066%) [12]. The juice from the fruit is used as antibiotics for the treatment of eye infections in Sierra Leone. The stems are used as chewing stick for oral hygeine in Nigeria [13]. The decoction or infusion of the stem has also been claimed to be used as aphrodisiac and anticarcinogen [14]. 

 Previous studies have reported that the alkaloidal content of *M. acuminata *stem was responsible for the antibacterial and antiinflammatory activities of the plant [15, 16]. Futhermore, a recent study by Yakubu et al. [17] have also validated the aphrodisiac claim of aqueous extract of *M. acuminata *root at 50, 100, and 200 mg/kg body weight in male rats. In addition, Yakubu et al. [12] have also reported that the aqueous extract of *M. acuminata* stem at the doses of 250, 500, and 1000 mg/kg body weight exhibited dose related androgenic and gonadotropic effects in male rats. Thus, it is logical to investigate the acclaimed aphrodisiac potential of the plant stem in a complete randomized design at the same doses used previously by Yakubu et al. [12]. 

 The present investigation was therefore undertaken to evaluate the aphrodisiac activity of aqueous extract of *M. acuminata *stem using the same doses (250, 500 and 1000 mg/kg body weight) that produced significant and dose related androgenic effects in male rats in our previous study [12]. This is with a view to validating the acclaimed use of the plant stem as sexual invigorator in the folk medicine of Nigeria.

## 2. Materials and Methods

### 2.1. Plant Material and Authentication

Samples of the plant obtained from herb sellers at Ijebu Ode, Nigeria, were authenticated by late Mr. Felix Usang of the Forestry Research Institute of Nigeria (FRIN), Ibadan, Nigeria, where a voucher specimen (FHI107644) was deposited.

### 2.2. Experimental Animals

Healthy, male rats (*Rattus norvegicus*), 2–2.5 months old weighing between 200–220 g and females, 1.5–2 months old weighing between 155–165 g were obtained from the Animal Holding Unit of the Department of Biochemistry, University of Ilorin, Nigeria. The animals were housed in clean aluminum cages placed in well-ventilated house conditions (Temperature: 28–31°C; photoperiod: 12 h natural light and 12 h dark; humidity: 50–55%). They were also allowed unrestricted access to rat pellets (Bendel Feeds and Flour Mills Ltd., Ewu, Nigeria) and tap water.

### 2.3. Assay Kits

The testosterone enzyme immunoassay test kit was a product of Diagnostic Automation Inc., Calabasas, USA, while estradiol benzoate and progesterone were products of Sigma Chemical (St. Louis, USA) and Shalina Laboratories Mumbai, India, respectively.

### 2.4. Preparation of Extract

The stem of *M. acuminata *obtained after removing the bark was cut into pieces and oven-dried at 40°C until a constant weight was obtained. The dried pieces were then pulverized with an electric blender (Blender/Miller III, model MS-223, Taipei, Taiwan). A portion (200 g) of the powder was extracted in 2 L of cold distilled water for 48 h at room temperature with constant shaking on a Stuart Scientific Orbital Shaker, SO1, Stone, UK. The extract was filtered with Whatman No. 1 filter paper (Maidstone, Kent, UK) and lyophilized to give 5.48 g. The resultant yield was reconstituted in distilled water to give the required doses of 250, 500 and 1000 mg/kg body weight used in this study. The doses were as used in our previous study on the androgenic potential of aqueous extract of *M. acuminata *stem [12].

### 2.5. Animal Grouping and Extract Administration

A total of 120 animals made up of equal number of male and female rats were used for this study in a complete randomized design. Sixty male rats were grouped into 4 (A–D) consisting of 15 animals each. Animals in group A (control) were orally administered, once daily with 1mL of distilled water (vehicle) using metal oropharyngeal cannula. Animals in groups B, C and D were treated with 250, 500 and 1000 mg/kg body weight of the extract, respectively, following dilution in 1 mL of the vehicle. Five male rats from each of the groups were monitored for sexual behaviour on day 1 (after a single dose), day 3 (after three doses, once daily) and day 5 (after five doses, once daily). The animals were handled humanely in accordance with the guidelines of European convention for the protection of vertebrate animals and other scientific purposes-ETS-123 [18].

### 2.6. Male Rat Sexual Behaviour Test Procedure

The male rats were trained with sexually receptive females, 3 times, for 4 days before the commencement of the experiment. The test was carried out between 19:00 and 22:00 h under a dim light. The female rats were made receptive following the sequential, subcutaneous administration of 10 *μ*g/100 g body weight of estradiol benzoate and 0.5 mg/100 g body weight of progesterone, 48 h and 4 h, respectively, prior to pairing. This treatment assures intense proceptivity and receptivity [19]. The receptive female was introduced to the male after a 30 min adaptation period in a plastic cage of dimensions 33.0 cm × 20.5 cm × 19.0 cm. The receptive female and male rats were observed from the cage side for proceptive and precopulatory behaviours, respectively. Five rats from each of the groups were monitored for sexual behaviour for 30 min observatory period, after their daily doses on days 1, 3, and 5. Adopting the standard procedures of Agmo [19] and Gauthaman et al. [20], the following male sexual behaviour indices were recorded or calculated for the observatory period: mount frequency (MF), (the number of times the male assumed copulatory position but failed to achieve intromission-characterized by lifting of the male's forebody over the hindquarter of the female and clasping her flanks with his forepaw); intromission frequency (IF), (the number of vaginal penetration made by the male); ejaculation frequency (EF), (the number of times there was expulsion of semen by the males after vaginal penetration-characterized by rhythmic contraction of the posterior abdomen). The female rats were also observed for the presence of vaginal plug. In addition, other standard parameters of sexual behaviour obtained through manual data acquisition using stopwatch included mount latency (ML), (the time from the introduction of the female until first mount by the male); intromission latency (IL), (the time from the introduction of the female until the first intromission by the male-usually characterized by pelvic thrusting and springing dismount; ejaculation latency (EL), (the time from the first intromission until ejaculation-usually characterized by longer, deeper pelvic thrusting and slow dismount followed by a period of reduced activity; and postejaculatory interval (PEI), (the time interval from ejaculation to intromission of the next series). Some additional male sexual behaviour parameters computed include: % index of libido = (number mated/number paired) × 100; % mounted = (number mounted/number paired) × 100; % intromitted = (number of rats that intromitted/number paired) × 100; % ejaculated = (number of rats that ejaculated/number paired) × 100; copulatory efficiency = (number of intromissions/number of mounts) × 100; intercopulatory efficiency = average time between intromissions.

### 2.7. Preparation of Serum

The animals were anaesthetized in a jar containing cotton wool soaked in diethyl ether. When rats became unconscious, their neck region was quickly cleared of fur and skin to expose their internal jugular veins. The veins were slightly displaced (to prevent contamination of the blood with interstitial fluid) after which they were cut sharply with a sterile blade. The rats were then held head downwards, allowed to bleed into clean, dry centrifuge tubes. The blood samples were allowed to clot for 10 min at room temperature and subsequently centrifuged at 224 × g for 10 min with Uniscope Laboratory Centrifuge (model SM800B, Surgifriend Medicals, Essex, England). The sera were aspirated with Pasteur pipette and used for the determination of testosterone concentration within 12 hours of preparation.

### 2.8. Determination of Serum Testosterone Concentration

The serum testosterone concentration of the animals was determined using the procedure outlined in the manufacturer's instruction manual. This was based on the principle of competitive binding between testosterone in the test specimen (serum) and testosterone-HRP conjugate for a constant amount of rabbit antitestosterone [21]. The assay procedure entailed dispensing 10 *μ*L of testosterone reference standards (0, 0.1, 0.5, 2.0, 6.0 and 18.0 ng/mL), serum (diluted ×5) and testosterone controls 1 and 2 into a Goat Anti-Rabbit IgG-coated microtitre wells (96 wells) after which 100 *μ*L of testosterone-HRP conjugate reagent (blue colour) and 50 *μ*L of rabbit antitestosterone reagent were separately dispensed into each well. The resulting solution was thoroughly mixed for 30 seconds and incubated at 37°C for 90 minutes. This was to allow a fixed amount of HRP-labelled testosterone to compete with the endogenous testosterone in the standard, sample, or quality control serum for a fixed number of binding sites of the specific testosterone antibody (since the amount of testosterone peroxidase conjugate immunologically bound to the well progressively decreases as the concentration of testosterone in the specimen increases). The microwells were rinsed and flicked 5 times with distilled water (to remove the unbound testosterone peroxidase conjugate) before dispensing 100 *μ*L of TMB reagent into each well. The resulting solution was mixed gently for 5 seconds. This was later incubated at room temperature for another 20 minutes for blue colour to develop. The colour development was stopped with the addition of 100 *μ*L of Stop Solution (1N HCl) to each well and when gently mixed, the colour changed from blue to yellow. The absorbance was read within 15 mins at 450 nm with a microtitre well reader. The intensity of the colour formed was proportional to the amount of enzyme present and was inversely proportional to the amount of unlabelled testosterone in the sample. The testosterone concentration in the serum of the animals was calculated from a calibration curve (obtained by plotting the concentration of the standard against the absorbance) using the following expression:

(1) $$\text{Testosterone}\;\text{concentration}\;\left(\text{ng}/\text{mL}\right)=\;C_{s}\times F,$$

where *C*_*s*_ is Corresponding testosterone concentration from the calibration curve and *F* is Dilution factor.

### 2.9. Statistical Analysis

Data were expressed as the mean of five replicates ± SD. Means were analyzed using a one-way analysis of variance (ANOVA) and complemented with Student's *t*-test. Post test analysis was carried out using Duncan Multiple Range Test and Tukey's Multiple Comparison Test to determine significant differences in all the parameters. All the statistical analyses were done using SPSS, Version 15.0 (SPSS Inc., Chicago, IL, USA) while the graphs were plotted with Excel Programme. Differences with values of *P* < .05 were considered statistically significant [22].

## 3. Results

Several female proceptive and male precopulatory behaviour parameters were observed from the cage side when the extract-treated male rats were introduced to the receptive female rats. The proceptive behaviour displayed by the female rats included ear-wiggling characterized by a rapid anteroposterior vibration of the ears, a short run where the female rats suddenly stops and present her posterior to the male rats (darting) and a short jump with stiff legs followed by immobility and presentation (hopping). The male rats, upon introduction, responded with immediate advances towards the females and displayed precopulatory behaviour such as chasing, anogenital sniffing which eventually culminated into mounting. Lordosis was also displayed by the receptive female rats before, at the beginning and during the mounts. There was genital toileting after every mount that resulted in intromission. The extract produced no sedative effect on the male rats since none of the animals showed evidence of tiredness throughout the observatory period. Similarly, dot receptivity was not displayed by any of the female rats used in this study.

**Figure 1:**
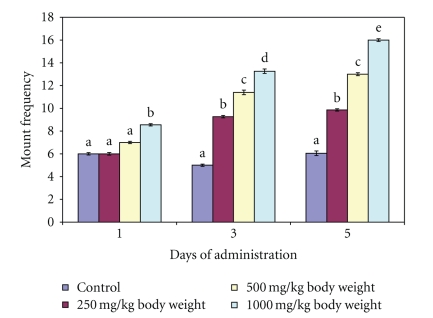
Effect of administration of aqueous extract of *Massularia acuminata* stem on mount frequency of male rats. Values are means of five replicates ± SD; bars carrying letters, b, c, d and e, different from their controls, a, on each day (i.e., days 1, 3 and 5) are significantly different at *P* < .05; bars carrying letters different for the same dose group at different days are significantly different at *P* < .05; bars carrying letters different from other dose groups are significantly different at *P* < .05.

**Figure 2:**
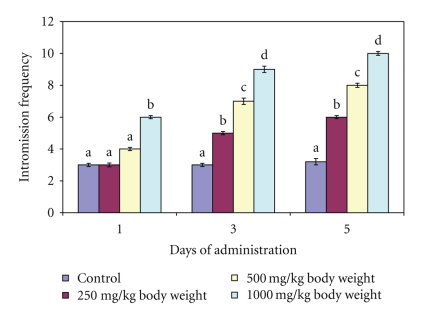
Effect of administration of aqueous extract of *Massularia acuminata* stem on intromission frequency of male rats. Values are means of five replicates ± SD; bars carrying letters, b, c, and d, different from their controls, a, on each day (i.e., days 1, 3 and 5) are significantly different at *P* < .05; bars carrying letters different for the same dose group at different days are significantly different at *P* < .05; bars carrying letters different from other dose groups are significantly different at *P* < .05.

 The aqueous extract of *M. acuminata *stem at 250 and 500 mg/kg body weight did not have any significant effect (*P* > .05) on the MF and IF following the administration of single dose of the extract (Day 1) whereas at 1000 mg/kg body weight, both MF and IF were increased compared to the distilled water-administered control. However, both MF and IF increased significantly (*P* < .05) on day 3 and day 5 for the 250, 500 and 1000 mg/kg body weight (Figures 1 and 2). The MF of the extract-treated animals was not the same as IF for each dose and day of observation. 

**Figure 3:**
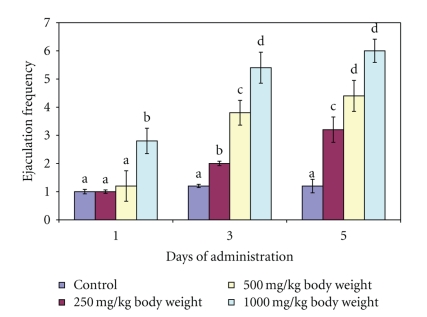
Effect of administration of aqueous extract of *Massularia acuminata* stem on ejaculation frequency of male rats. Values are means of five replicates ± SD; bars carrying letters, b, c, and d, different from their controls, a, on each day (i.e., days 1, 3 and 5) are significantly different at *P* < .05; bars carrying letters different for the same dose group at different days are significantly different at *P* < .05; bars carrying letters different from other dose groups are significantly different at *P* < .05.

 Administration of single dose of aqueous extract of *M. acuminata *stem at 250 and 500 mg/kg body weight did not significantly (*P* > .05) affect the EF of the male rats whereas at 1000 mg/kg body weight, the EF increased significantly (*P* < .05). At day 3 and day 5, the extract at all the doses investigated significantly (*P* < .05) increased EF in the animals (Figure 3). There was vaginal plug in the female's vagina after ejaculation.

**Figure 4:**
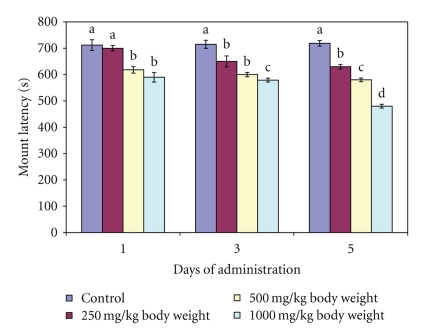
Effect of administration of aqueous extract of *Massularia acuminata* stem on mount latency of male rats. Values are means of five replicates ± SD; bars carrying letters, b, c, and d, different from their controls, a, on each day (i.e., days 1, 3 and 5) are significantly different at *P* < .05; bars carrying letters different for the same dose group at different days are significantly different at *P* < .05; bars carrying letters different from other dose groups are significantly different at *P* < .05.

**Figure 5:**
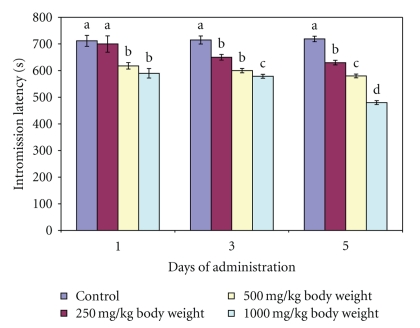
Effect of administration of aqueous extract of *Massularia acuminata* stem on intromission latency of male rats. Values are means of five replicates ± SD; bars carrying letters, b, c, and d, different from their controls, a, on each day (i.e., days 1, 3 and 5) are significantly different at *P* < .05; bars carrying letters different for the same dose group at different days are significantly different at *P* < .05; bars carrying letters different from other dose groups are significantly different at *P* < .05.

 In contrast, both ML and IL at 250 mg/kg body weight compared favourably (*P* > .05) with the distilled water-administered control. Further administration of all the doses of the extract for 3 and 5 days significantly (*P* < .05) decreased both the ML and IL of the animals (Figures 4 and 5). 

**Figure 6:**
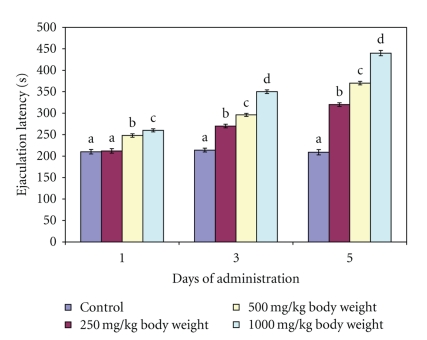
Effect of administration of aqueous extract of *Massularia acuminata* stem on ejaculation latency of male rats. Values are means of five replicates ± SD; bars carrying letters, b, c, and d, different from their controls, a, on each day (i.e., days 1, 3 and 5) are significantly different at *P* < .05; bars carrying letters different for the same dose group at different days are significantly different at *P* < .05; bars carrying letters different from other dose groups are significantly different at *P* < .05.

**Figure 7:**
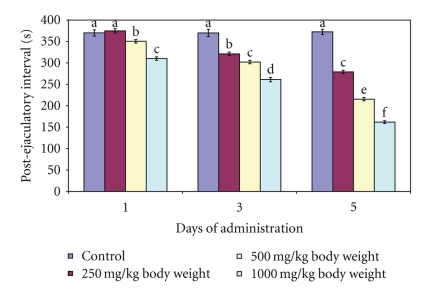
Effect of administration of aqueous extract of *Massularia acuminata* stem on postejaculatory interval of male rats. Values are means of five replicates ± SD; bars carrying letters, b, c, d, e and f, different from their controls, a, on each day (i.e., days 1, 3 and 5) are significantly different at *P* < .05; bars carrying letters different for the same dose group at different days are significantly different at *P* < .05; bars carrying letters different from other dose groups are significantly different at *P* < .05.

 The extract produced contrasting effects on the EL and PEI of the male rats. For example, whereas the EL increased following single dose of 500 and 1000 mg/kg body weight, the PEI decreased at these doses. The single administration of 250 mg/kg body weight for both the EL and PEI were not significantly altered compared to the untreated control (Figures 6 and 7). Further administration of the extract at all the doses for 3 and 5 days increased the EL in dose related manner (Figure 6) whereas the PEI decreased in a dose related manner (Figure 7). The alterations in these parameters were statistically significant (*P* < .05). 

 The computed male sexual behaviour parameters which included percentages of index of libido, mounted, intromitted, ejaculated and copulatory efficiency were higher in the extract-treated animals compared to the untreated distilled water control animals (Table 1). In contrast, the extract reduced the intercopulatory interval of the animals in dose related manner compared to the distilled water-administered control animals. The decreases observed were statistically significant (*P* < .05). 

 The aqueous extract of *M. acuminata *stem at 250 mg/kg body weight on day 1 and day 3 did not have any effect on the serum testosterone content of the animals compared to the untreated control animals. However, the 500 and 1000 mg/kg body weight increased the concentration of the hormone on day 1 and day 3. In addition, these increases were extended to all the doses on day 5. By the end of the experimental period, the highest dose group of the extract (1000 mg/kg body weight) had increased the testosterone concentration in the serum of the animals by 1.8 fold of the control (Table 2). The increases observed were statistically significant (*P* < .05). 

## 4. Discussion

The search for aphrodisiacs that can increase libido, potency and sexual pleasure dates back to millennia. Various substances of animal and plant origin have been used in folk medicine of different cultures as aphrodisiacs, some of which have been identified pharmacologically to exert their effects on the hypothalamic-pituitary-testicular axis. Furthermore, ancient literature alluded to the use of numerous plants/preparations including *M. acuminata *as sex enhancer without any scientific evidence [14]. To understand the scientific reasons behind these folk claims, we investigated the effects of aqueous extract of *M. acuminata *stem in this study. In this investigation, treatment of the male rats with the aqueous extract of *M. acuminata *stem enhanced the sexual behaviour of the male rats with 500 and 1000 mg/kg body weight producing better results than the 250 mg/kg body weight. These sexual behaviours were preceded with proceptive and precopulatory behaviours in the animals. For example, the ear-wiggling, darting, hopping and lordosis by the receptive female rats in this study implied intense proceptivity and receptivity whereas the precopulatory behaviour by the extract-treated male rats also suggested that the animals were generally aroused. The pursuit of the female animals (the males running behind the female animals in close contact) suggested imminent copulation. 

 Mount Frequency and Intromission Frequency are useful indices of vigour, libido and potency. While the number of mount (MF) reflects sexual motivation, increase in the number of intromission (IF) shows the efficiency of erection, penile orientation and the ease by which ejaculatory reflexes are activated [19]. Therefore, the increase in MF and IF following the administration of aqueous extract of *M. acuminata *stem at 1000 mg/kg body weight on day 1 and subsequently at all the doses on other days of observation suggests enhanced libido [6]. Such enhancement of libido might have arisen from increase in the number of concentrations of several anterior pituitary hormones and serum testosterone, which in turn stimulated dopamine receptor synthesis and sexual behaviour [23]. This sexual behaviour may also be due to androgenic and gonadotropic activities of *M. acuminata *stem in male rats reported in our previous study [12]. It may therefore be logical to attribute these behaviours to flavonoid and or saponin constituents of the plant since they have been reported to alter androgen levels [20]. The alteration in the androgen level was supported by the increase in testosterone content of the animals in the present study. Furthermore, since intromission is not possible without adequate erection and coordinated activity of penile muscles [19], the increase in IF by the extract in this study suggests that the mechanism of penile erection was activated. Therefore, aqueous extract of *M. acuminata *stem may increase potency by allowing or sustaining erection. Various phytochemicals have been reported to affect penile erection by different mechanisms. For example, alkaloids have been shown to have ergogenic properties by inducing vasodilation of the blood vessels which consequently result in erection [19]. In addition, Kim et al. [10] also reported that the saponin content of *Panax gingseng *acting as a nitric oxide donor may induce the relaxation of smooth muscle *Corpus cavernosum* through the L-arginine/nitric oxide pathway. Furthermore, the aphrodisiac property of *P. gingseng* root has also been attributed to enhance acetylcholine-induced and transmural nerve stimulation-activated relaxation associated with increased tissue cyclic guanosine monophosphate. Therefore, the enhanced IF by the extract of *M. acuminata *stem which may be associated with the alkaloid and or saponins content of the plant via these mechanisms may await further studies. The disparity, in the values of MF and IF in this study suggests that it was not every mount by the male rats that resulted in intromission. Similarly, the increase in ejaculation frequency by the extract of *M. acuminata *stem at 1000 mg/kg body weight on day 1 and by all the doses at days 3 and 5 is an indication of enhanced aphrodisiac effect of the plant. The presence of plug in the vagina of the female rats indicated that ejaculation occurred. This was further complemented by the genital toileting observed in the male rats.

 Mount latency and intromission latency are indicators of sexual motivation. ML and IL are inversely proportional to sexual motivation. Therefore, the decrease in the mount and intromission latencies observed at the doses of 500 and 1000 mg/kg body weight of the extract on day 1 as well as at all the doses investigated on days 3 and 5 in this study might imply stimulation of sexual motivation and arousability. It may also be an indication of enhanced sexual appetitive behaviour in the male rats. All these further support the sexual function improving effect of the extract at these doses. Furthermore, the prolonged ejaculation latency by the 500 and 1000 mg/kg body of aqueous extract of *M. acuminata *stem on day 1 and at all the doses on days 3 and 5 is an indication that copulatory performance in the animals was enhanced. It may also imply prolongation in the duration of coitus. In addition, the display of pelvic thrusting during intromission and ejaculation by the extract-treated animals in this study further indicated that the male copulatory organ was in contact with the vaginal orifice which might have activated or strengthened lordosis in the female rats [19].

 The post ejaculatory interval is considered an index of potency, libido and the rate of recovery from exhaustion after first series of mating [6]. A post ejaculatory interval of more than 5400 sec indicates that the male is sexually exhausted and the intensity of sexual behaviour will be reduced in subsequent mating [19]. Therefore, the significantly decreased post ejaculatory interval at 500 and 1000 mg/kg body of aqueous extract of *M. acuminata *stem on day 1 and at all the doses on days 3 and 5 may be attributed to enhanced potency and libido or less exhaustion in the first series of mating or both, more so, since the values of PEI obtained in this study are not up to or in any way close to the 90 min cut-off. In addition, the higher values of the computed male rat sexual behaviour parameters following treatment with the extract when compared with the distilled water-administered control animals are indications of significant and sustained increase in sexual activity [24].

 Many plants with medicinal properties are effective as aphrodisiac through mechanisms such as vasodilation, generation of nitric oxide, elevation of androgens and gonadotropins. It has also been documented that sexual behaviour and erection are dependent on androgen which may act through central and peripheral mechanisms [25]. Treatments that alter the concentration of circulating sex hormones may also modify sexual behaviour. Clinical data on testosterone also suggest that a slight increase in the levels of the hormone in adult males results in a moderate but significant increase in sexual desire and libido [26]. Therefore, the increase in serum testosterone concentration by the aqueous extract of *M. acuminata *stem at 500 and 1000 mg/kg body weight on days 1 and 3 as well as at all the doses on day 5 might be responsible for the enhanced sexual behaviour in the animals. Dehydroepiandrosterone (DHEA), a major circulating steroid in the plasma, and a common precursor for both androgens and estrogens, acts centrally as a gamma amino butyric acid antagonist to facilitate sexual function [27]. Although, the levels of DHEA was not determined in this study, a possible increase in DHEA, its subsequent conversions to testosterone and its metabolites may account for the observed enhanced masculine behaviour in this study. The involvement of steroidal glycosides (saponins) in the biosynthesis of DHEA [28] may therefore boost the level of testosterone in the body as well as trigger libido enhancing effect observed in this study. In addition, the presence of flavonoids in the extract which has been implicated to have a role in altering androgen levels may also be responsible for the enhanced male sexual behaviour in this study [29]. Therefore, the raised level of testosterone in this study could be attributed not only to the enhanced sexual performance by the male rats but also to the antioxidant as well as androgenic and gonadotropic properties of the flavonoids and saponins reportedly present in the plant extract in our previous study [12]. 

**Figure 8:**
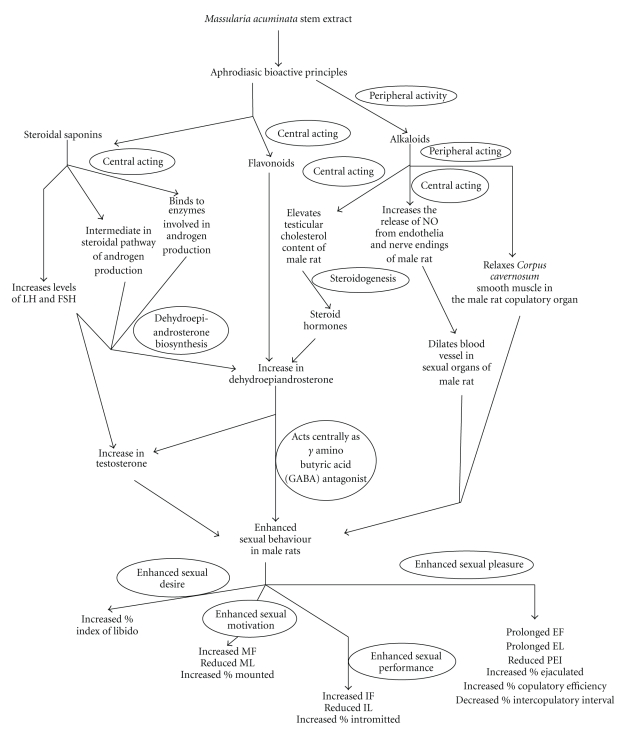
Possible mode of action of *Massularia accuminata* stem as sexual invigorator in male rats.

 Saponins and flavonoids in the aqueous extract of this plant might have assisted in stimulating increase in the body natural endogenous testosterone levels probably by raising the level of luteinizing hormones, which translated into the male sexual competence observed in this study. The steroidal nature of saponins may facilitate its role as an intermediary in the steroidal pathway of androgen production [28]. Saponins may bind to hormone receptors which may result in conformational change that will enhance the physiological function of the hormone or bind to enzymes that are involved in the synthesis of such hormones and thus enhance its production as observed in this study. Furthermore, the well known antioxidant property of flavonoids, one of the constituents of the plant extract which has been reported to alter androgen levels in animals may also contribute to the aphrodisiac effect in this study. In addition, the presence of alkaloids which have been reported to have ergogenic properties may act centrally either by inducing vasodilation of the blood vessels through the production of nitric oxide and thus allowing erection or stimulates steroidogenesis in the testes of the animals. Alkaloids may also act peripherally by relaxing *Corpus cavernosum *smooth muscle in the copulatory organ of the male rats. The possible hypothetical interaction of the plant and or its components in enhancing libido, sustaining erection and enhancing sensory experience during coitus in male rats is depicted in Figure 8. Therefore, it is possible that the active principle(s) contained in the extract might have crossed the blood-brain barrier of the animals to exert its aphrodisiac effect on the hypothalamic-pituitary-testicular axis. 

 Overall, our results have revealed that the aqueous extract of *Massularia acuminata *stem at the doses of 500 and 1000 mg/kg body weight could be used as a stimulator of sexual behaviour in male rats. This study thus supports the acclaimed aphrodisiac use of the plant in folk medicine of Nigeria. The data obtained revealed that the action of *M. acuminata *extract was due to the influence on both sexual arousal and performance. The aphrodisiac effect of the plant extract may be due to the presence of alkaloids, saponins and/or flavonoids through a multitude of central and peripheral means. Work is in progress on the isolation and characterization of the aphrodisiac principle(s) in the plant extract, the actual mechanism of action as well as the toxicity risks of the crude extract and bioactive agent(s).

## Figures and Tables

**Table 1:** Effect of aqueous extract of *Massularia acuminata* stem for 5 days on computed male rat sexual behaviour parameters.

Parameters	Extract (mg/kg body weight)			
	Control	250	500	1000
Index of Libido (%)	60	80	100	100
% Mounted	60	100	100	100
% Intromitted	20	60	80	100
% Ejaculated	10	40	40	60
Copulatory efficiency (%)	52.89	60.85	61.54	62.50
Intercopulatory interval (sec)	720.00 ± 36.00^(a)^	590.00 ± 20.00^(b)^	106.00 ± 15.00^(c)^	72.00 ± 6.00^(d)^

Values are means of five replicates ± SD; superscripts, b, c, and d, different from the control, a, for the intercopulatory interval are significantly different (*P* < .05).

**Table 2:** Effect of aqueous extract of *Massularia acuminata *stem on serum testosterone concentrations of male rats.

Days/ Doses (mg/kg body weight)	Serum Testosterone Concentration (nM/L)		
	Days		
	1	3	5
Control	2.20 ± 0.13^(a)^	2.22 ± 0.09^(a)^	2.12 ± 0.22^(a)^
250	2.22 ± 0.10^(a)^	2.31 ± 0.06^(a)^	2.48 ± 0.04^(b)^
500	2.50 ± 0.05^(b)^	2.61 ± 0.10^(b)^	2.78 ± 0.03^(b)^
1000	2.62 ± 0.07^(c)^	3.08 ± 0.04^(c)^	4.61 ± 0.07^(c)^

Values are means of five replicates ± SD; superscripts, b and c, different from the control, a, for each day and for each parameter are significantly different (*P* < .05).
